# Cancer Therapy: A Continuance of Health Burden

**DOI:** 10.4021/wjon581e

**Published:** 2012-10-28

**Authors:** Sowmya Kasetty, Samar Khan, Sudheendra U Shridhar, Sandeep Gupta, Manisha Tijare, Shreenivas Kallianpur, T Raju Ragavendra

**Affiliations:** a Department of Oral Pathology, Peoples College of Dental Sciences and Research Centre, Bhopal-462037, MadhyaPradesh, India

**Keywords:** Cancer, Therapy, Health burden

## Abstract

**Background:**

Cancer diagnosis coupled with emotional impact converge to create one of the most difficult physical and emotional periods of life. Cancer treatment causes plethora of short and long term complications which can be so debilitating that patient may interrupt treatment. Pretreatment oral assessment and supportive oral care during and after cancer therapy can increase quality of life and supportive care costs.

**Methods:**

Study was conducted on 189 patients (86: head and neck cancer cases, group I and 103: other than head and neck cancer cases, group II) receiving cancer therapy. Patients were subjected to clinical assessment and findings were recorded in specially designed proforma and complete oral (objective and subjective) and constitutional findings were recorded.

**Results:**

Among the patients undergoing chemotherapy in both groups, prevalence of oral findings was found to be highest with methotrexate whereas constitutional symptoms was found to be highest with doxyrubicin. Whereas in radiotherapy patients subjective and objective oral symptoms increased from 10th - 30th fractionated dose of radiations and then subsequently decreased and constitutional symptoms were found to be consistent in all fractionated dosages with lowest at 50th fraction. Under combined chemo and radiotherapy patients, constitutional symptoms were highest than the oral findings.

**Conclusions:**

Cancer therapy can greatly damage the normal tissues and diminish patients quality of life and often leads to serious clinical sequelae. Therefore, therapy induced damage should be anticipated and prevented whenever possible and managed early.

## Introduction

Head and neck cancer treatment primarily involves three modalities namely surgery, radiotherapy and chemotherapy, administered alone or in combination. Chemotherapy along with surgery or radiotherapy for advanced disease causes a plethora of short and long-term oro and oropharyngeal squeal. These complications are complex, dynamic pathobiological processes that lower the quality of life and predispose patients to serious clinical disorders.

Most common adverse effects of these therapies includes mucositis, local or systemic infection, salivary gland dysfunction, taste alteration and pain, which later lead to secondary complications such as nutritional disorder, xerostomia or hemorrhage [1].

These treatment modalities not only destroy cancer cells but normal cells also and tissues with high rates of proliferation namely hair follicles, gastrointestinal tract and oral cavity [1-4].

Therapy based oral complications are due to high cellular turnover rates of the oral mucosa, a diverse and complex microflora, and trauma to oral tissues during normal oral function and often results in epithelial and glandular destruction and inflammation.

Complications are so debilitating that patients may tolerate only lower less-effective doses of therapy, may postpone treatments or will discontinue treatment entirely. Considerable number of studies has been reported demonstrating varied adverse effects on oral mucosa due to cancer therapy but very few articles are reported depicting individual effects of drug and fractionation of radiation therapy causing stomatotoxicity.

Thus the present study was conducted to evaluate the impact of cancer therapy regimens on individual well being and to establish relationship between chemotherapeutic drugs and oral side effects and also to evaluate relative effect of fractions of radiations on oral mucosa. Our objective was to characterize from the patient’s perspective, the consequences resulting from cancer therapy.

## Materials and Methods

Hospital based survey performed on 189 patients with the age range of 16 - 70 years who were undergoing chemo and radiotherapy at Jawaharlal Nehru Cancer Hospital and Research Centre, Bhopal (MP).

The study group comprised of 86 patients in group I (head and neck cancer cases: HNCC) and 103 patients in group II (other than head and neck cancer cases: OHNCC). Distribution of patients undertaking various treatments is given in Table 1. The cancer site predilection among group I was found to be highest in buccal mucosa, tongue and lowest in nasopharynx and in group II, breast cancer was highest followed by haemopoietic tumors and least being in testis and penis.

**Table 1:** Cases in Group I and II Undergoing Cancer Therapy

Groups	Description	Cancer Therapy	No. of cases
Group I (n = 86)	Head and Neck Cancer cases	Chemotherapy	22
		Radiotherapy	32
		Combined (Chemo and Radio) Therapy	32
Group II (n = 103)	Other Than Head and Neck Cancer cases	Chemotherapy	51
		Combined (Chemo and Radio) Therapy	52

Patients in each group were subjected to clinical assessment with prior consent from concerned authority followed by recording of findings in specially prepared performa to assess subjective and objective oral hard and soft tissue findings along with constitutional symptoms in response to chemo and radiotherapy (Table 2).

**Table 2:** List of Signs and Symptoms

Therapy complications	Signs and Symptoms
Subjective oral Findings	Xerostomia, dysphonia, trismus, taste alteration, halitosis and neuropathy.
Objective oral Findings	Ulcers, erythema, dysphagia, edema, infections, periodontal findings and hard tissue findings.
Constitutional symptoms	Anxiety, irritability, lacrimation, abdominal cramps, vomiting, chills/hot flashes, diaphoresis, diarrhea, joint pain and nausea.

Oral complications due to chemotherapy were analyzed with respect to most commonly administered chemotherapeutics drugs (cyclophosphamide, doxyrubicin, 5-fluorouracil, methotrexate, cisplatin, paclitaxel) in both group I and II. Radiotherapy associated complications were recorded correlating with fraction of radiation dose in group I alone and findings was also evaluated in patients undergoing combined therapy (chemo and radiotherapy) in both groups and results were tabulated in excel sheet for analysis.

## Results

**Figure 1:**
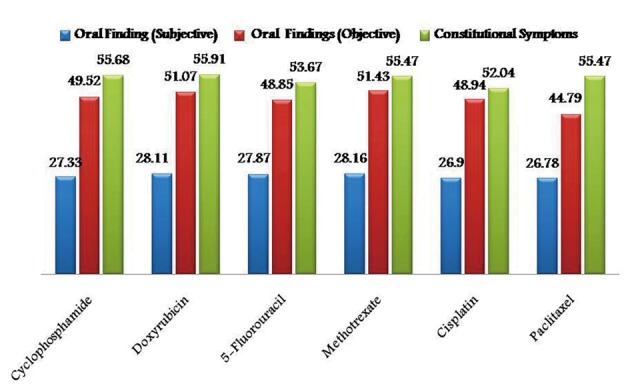
Graphical representation showing prevalence of adverse effects of chemotherapy in group I and II.

**Figure 2:**
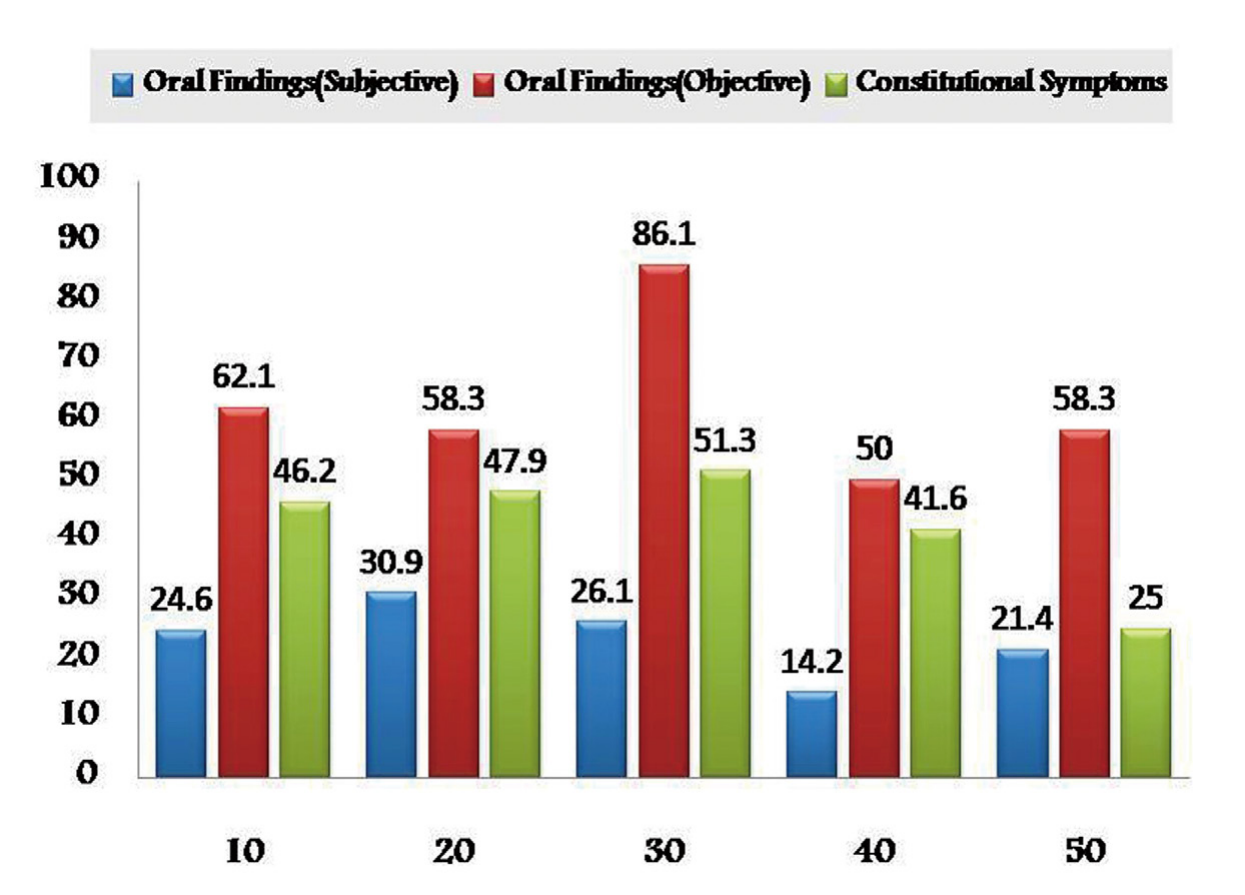
Graphical representation showing prevalence of adverse effects of radiotherapy in Group I.

As the chemotherapeutic drugs administrated were common to both the groups, results were tabulated together. Prevalence of subjective and objective oral findings in both the groups was found to be highest with methotrexate (28.16% and 51.43%) and lowest with Paclitaxel drug (26.78% and 44.79%) respectively. Whereas constitutional symptoms was found to be highest with doxyrubicin (55.91%) and minimum with cisplatin (52.04%) (Fig. 1). Whereas some cases of group I are treated with radiotherapy alone after surgical resection in such cases prevalence of subjective and objective oral symptoms increased from 10th - 30th fractionated dose of radiations and then subsequently decreased and found to be lowest at 50th fractionated dosage. Constitutional symptoms were found to be consistent in all fractionated dosages with lowest at 50th fraction (Fig. 2).

**Figure 3:**
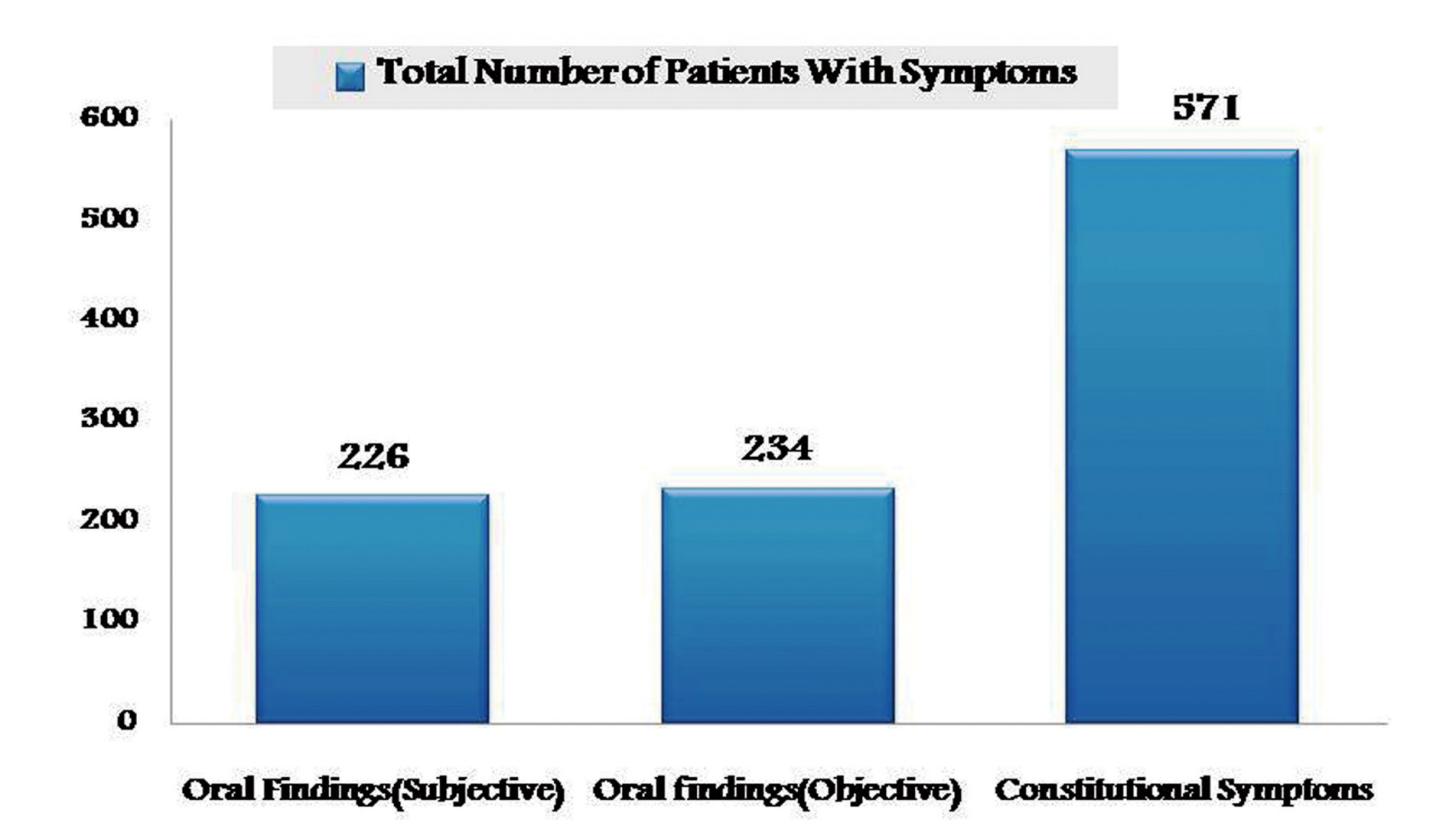
Graphical representation showing prevalence of adverse effects of combined therapy in group I and II.

Cases of regionally advanced disease in both group I and II after surgical intervention, have undergone combined chemo and radiotherapy in which constitutional symptoms were highest when compared to oral findings (Fig. 3). However there was no correlation between cancer site and the adverse effects of treatment.

## Discussion

Prevalence of oral complications from drug and radiation due to cancer therapy lies in the limited literature and potentially had unreported. Cancer therapy adverse effects results from two mechanism: a direct effect of the drug or irradiation on the oral mucosa (direct stomatotoxicity) or an indirect result of myelosupresion from drug or radiation therapy (indirect stomatotoxicity) [3]. The direct inhibitory effects of chemotherapy on DNA replication and mucosal cellular proliferation result in a reduction in the renewal capacity of the basal epithelium and results in mucosal atrophy, collagen breakdown, ulceration, thrombocytopenia and leukopenia disturbing the hemostatic and immune-mechanisms of the patient [5, 6-9]. Whereas the indirect side effects in turn are due to collateral impact upon the oral cavity such as, bone marrow suppression, loss of tissue immune cells, and loss of salivary protective elements [1].

These complications may result in significant morbidity, treatment delays, dose reductions, and nutritional deficiencies [5, 10-12]. The type of chemotherapeutic agents, the dosage, and the frequency of drug administration are important therapy related factors which causes stomatotoxicity [4, 5, 13, 14]. Chemotherapeutic agents that are commonly used in head and neck cancer and have a high potential for precipitating oral mucosal damage are alkylating agents such as cisplatin, busulfan, cyclophosphamide, procarbazine, and thiotepa; anthracyclines such as daunorubicin, doxorubicin, and epirubicin; antimetabolites such as cytosine arabinoside, hydroxyurea, 5-fluorouracil, methotrexate, 6-mercaptopurine, and 6-thioguanine; antibiotics such as actinomycin D, amsacrine, bleomycin, and mitomycin; vinca alkaloids such as paclitaxel, vinblastine, vincristine; and taxanes [15, 16]. The acute effects of chemotherapy upon the oral cavity include mucositis, infection, hemorrhage, xerostomia, neurological disorders, and nutritional deficiencies [5, 17-20]. It has been reported that oral mucositis is a significant complication of high-dose chemotherapy in cancer patients which supports this study [4]. As per the reviewed literature cisplatin and fluorouracil are the most common chemotherapeutic agent with maximum side effects [1] but in contrast to this, present study revealed maximum subjective and objective oral side effects with methotrexate in both the groups.

Trends in the treatment of HNCC have led to an increasing use of chemotherapy with RT and the development of more intense RT regimens [2]. Fractionation is the term used for radiation schedule and standard fractionation refers to radiation taken once/week. Schedules used to intensify treatment for more advanced tumours are known as accelerated fractionation or hyperfractionation. Most HNCC are treated with two Gy/ fraction delivered five times/week, up to a total dose of 64 - 70 Gy [21]. The severity of oral complications is related to the daily and total cumulative dose of radiation, the volume of irradiated tissue, and use of concurrent radiation-sensitising and mucositis-inducing chemotherapeutic drugs [7, 8, 22-25].

Effects of tumorcidal doses of radiation on healthy oral mucosa are divided into acute type, which occurs during treatment or shortly afterwards (2 - 3 weeks) and chronic types may occur months or even years after therapy [26].

Previous studies reveal occurrence of oral mucositis in 97% and 100% of HNCC receiving conventional fractionated and altered fractionation RT radiotherapy respectively, which is in accordance with our study. In this study, only group I patients received radiotherapy without chemotherapeutic drugs after surgical intervention and showed increasing incidence of oral side effects from 10th - 30th fractions of radiation, whereas it decreased with advancing fractions this finding can be substantiated by the fact that as RT continues, a steady state between death and regeneration of mucosal cells could occur because surviving cells are produced at an increased rate.

Combined chemo and radio therapy is now considered standard for most locally advanced tumors in both the cancer groups. Chemotherapy can be given prior to radiation therapy or alongside with radiation therapy. The toxicities of this combined therapy are essentially the same as with radiation alone but they develop more rapidly and severe, when they reach maximum level and these patients will be more immunosuppressed than those receiving radiation therapy alone. Therefore suspicion of oral infection should be high which includes odontogenic, candidal and herpes simplex infections. However in contrast, the present study showed high predilection for constitutional symptoms when compared to oral findings in both group I and II patients. So to this end the study illustrate the debilitating nature of mucosal injury in patients with cancer therapy and the need for new and effective therapies for oropharyngeal mucositis.

### Conclusion

Cancer therapy though cures the disease, can also greatly damage oral tissues and cause other systemic adverse effects. So a standard assessment tools and optimal prophylactic or therapeutic agents, emphasis on frequent and meticulous oral care are important for reducing the severity of oral complications. Adverse effects may be minimized by introducing prior biological response modifiers, cytoprotective drugs, tissue-sparing radiation technique and surgical advances.
